# Supplementary material for: Analysis of PPARγ Signaling Activity in Psoriasis
Source: Int J Mol Sci. 2021 Aug 10;22(16):8603. doi: 10.3390/ijms22168603 (PMC8395241; doi:10.3390/ijms22168603)
Supplement: Supplementary file 1 [file ijms-22-08603-s001.zip › Supplemental materials_Analysis of PPARg signaling activity in psoriasis/Pathway models/Models images and html files/PPARG inhibits phenotypes assotiated with psoriasis/244951.html]

T-cell infiltration


# Disease T-cell infiltration

|  |  |
| --- | --- |
| URN | urn:agi-disease:T-cell%20infiltration |
| Total Entities | 1 |
| Connectivity | 1281 |
| Name | T-cell infiltration |

---

|  |  |
| --- | --- |
| ChildConcepts | cytotoxic T-cell infiltration |

---

|  |  |
| --- | --- |
| Pathway | PPARG inhibits phenotypes assotiated with psoriasis |
|  | efects selected |

---

|  |  |
| --- | --- |
| MedScan ID | 9018075 |

---

|  |  |
| --- | --- |
| Alias | T-lymphocyte infiltration |
|  | infiltration by T-lymphocytes |
|  | infiltration by T-cells |
|  | T-cell infiltration |
|  | T-lymphoblast infiltration |

---

|  |  |
| --- | --- |
| CellType | T-cell {CellType urn:agi-ncimcelltype:C0039194} |
|  | T-cell |

---

|  |  |
| --- | --- |
| CellProcess | cell infiltration {CellProcess urn:agi-cellproc:cell%20infiltration} |

---
